# Evaluation of the Antibacterial Activity of Patchouli Oil

**Published:** 2013

**Authors:** Xian Yang, Xue Zhang, Shui-Ping Yang, Wei-Qi Liu

**Affiliations:** a *College of Resources and Environment, Southwest University, Chongqing, 400716, P.R. China.*; b *Chongqing Academy of Chinese Materia Medica, Chongqing 400065, P.R. China.*; c *College of Bioengineering, Chongqing University, Chongqing 400044, P.R. China. *

**Keywords:** Patchouli oil, Molecular docking, Scoring function, Antibiotic experiment in vitro, MIC and MBC

## Abstract

In the present study, the antimicrobial tests of patchouli oil were studied by using molecular docking technology and antimicrobial test in vitro. Five biological macromolecule enzymes, required by the bacteria in the process of biosynthesis were selected as target molecules. Five antibiotics benzylpenicillin, sulfadiazine, trimethoprim, rifampicin and ciprofloxacin, which are generally acknowledged as antibacterial drugs, were selected as reference compounds. The 3 three-dimensional (3D) structures of the 5 reference compounds and 26 compounds from patchouli oil were established by using surflex-dock software (8.1). And the 3D structures of five biological macromolecule enzymes derived from Protein Data Bank (PDB). Molecular docking was carried out between the 31 chemical compounds (ligands) and the 5 enzymes (receptors) by using surflex-dock function. Furthermore, the antibacterial effects of 31 chemical compounds were investigated by the scoring function after molecular docking was completed. By comparing the scoring result of 26 compounds in patchouli oil with 5 compared components, we inferred antibacterial activity in about 26 compounds in patchouli oil. On the other hand, six frequently-used pathogenic bacteria were selected for antimicrobial test in vitro, patchouli oil and its two major compounds: (-)-patchouli alcohol and pogostone, which their contents exceeded 60% in patchouli oil samples, were selected antibacterial agents. Minimum inhibitory concentration (MIC) and Minimum bactericidal concentration (MBC) were also determined. Molecular docking technology and antimicrobial test in vitro proved that patchouli oil had strong antimicrobial effects. Particularly, pogostone and (-)-patchouli alcohol have potent antimicrobial activity.

## Introduction

The method of simulating a geometric model of molecular and intermolecular forces by chemometrics methods in order to identify and forecast receptor-ligand complex structure is called molecular docking. This technique has become an authentic procedure in drug discovery studies. It was originally proposed when chemical problems were encountered in the biological system which were based on the numerator level at that time. As early as 1789, E.Fisher used “key and lock” as a metaphor for enzyme-substrate in his paper, and he named it identification (1). At the same time, the receptor theory introduced by Langley proposed that most drugs must combine with some particular molecules on the cell membrane, and these particular molecules were named as receptor (2). Thus, the concept of receptor in receptor theory and in molecular docking is essentially the same which lays theoretical foundation for molecular docking approach. Molecular docking techniques have shown great promise as a new tool in the discovery of novel small molecule drugs for targeting proteins (3, 4).

Patchouli [*Pogostemon cablin *(Blanco) Benth.] is a Traditional Chinese Medicine (TCM) that has been widely used in Philippines, Malaysia, India and China. The dry leaves of patchouli on steam distillation yield an essential oil called the patchouli oil. Patchouli oil is hence an important ingredient in many fine fragrance products such as perfumes, as well as in soaps and cosmetic products (5). Results of High Performance Thin Layer Chromatography (HPTLC) studies indicated that the ethyl acetate extract of *Pogostemon parviflorus *leaves included triterpenes, as 10 and 14 peaks of ultra violet (UV) absorption were observed in 254 nm and 366 nm, respectively. Hence, triterpenes may be responsible for antidermatophytic activity of this plant (6). It is also on the FDA’s (Food and Drug Administration) list of substances approved for human consumption, in section 172.510, as a natural additive for food flavoring (7). 

Moreover, patchouli oil in the plant is widely used in TCM as it offers various types of pharmacological activities (7). It has also been reported to strengthen the immune activity and resistance to bacterial action *et al*. (8). The composition of the patchouli oil is complex like many essential oils, which consist of the major components such as patchoulol alcohol and pogostone etc. The mechanism of action of major pharmacologic components in patchouli oil as an antibacterial agent has not been reported. To identify the possible biochemical pathways involved and to assess the therapeutic potential of patchouli oil in bacterial infection, we evaluated its effects by using molecular docking technology and antibacterial activity *in-vitro*. 

In this study, 5 biological macromolecule enzymes: Penicillin binding proteins (PBPs), dihydrofolate synthetase (DHPS), dihydrofolate reductase (DHFR), DNA gyrase and RNA polymerase, which were needed by bacteria in the process of biosynthesis, were selected as target molecules. Additionally, 5 antibiotics, benzylpenicillin (acts on PBPs), sulfadiazine (acts on DHPS), trimethoprim (acts on DHFR), ciprofloxacin (acts on DNA gyrase) and rifampicin (acts on RNA polymerase), which are generally known as very good antibacterial drugs, were selected as reference compounds. The 3D structures of the 5 reference compounds and 26 compounds of patchouli oil were built by using the surflex-dock software (8.1) which is a molecular docking software developed by professor Ajay N.Jain (9-11) and the 3D structures of 5 biological macromolecule enzymes were derived from Protein Data Bank (PDB) (12). Molecular docking was carried out between the 31 chemical compounds (ligands) and the 5 enzymes (receptors) by using surflex-dock function. Furthermore, the antibacterial effects of 31 chemical compounds were investigated by the scoring function after molecular docking was completed. By comparing the scoring results of 26 compounds of patchouli oil with 5 compared components, we could infer antibacterial activity of 26 compounds of patchouli oil.

On the other hand, six frequently-used pathogenic bacteria (*Escherichia coli*, *Pseudomonas aeruginosa*, *Bacillus proteus*, *Shigella dysenteriae*, *Typhoid bacillus*, *Staphylococcus aureus*) were selected for testing antibacterial activity in vitro, patchouli oil and its two major compounds: (-)-patchouli alcohol and pogostone, exceeding over 60% (g/g) in patchouli oil samples (13), were selected. Minimum inhibitory concentration (MIC) and Minimum bactericidal concentration (MBC) were deremined in order to verify the result of molecular docking.

## Experimental

*Materials*

Patchouli samples were collected from Guangdong, China in October 2010 ,. Professor Bo-Chu Wang, College of Bioengineering of Chongqing University, identified the raw medicinal herbs, and the voucher specimens were deposited at the Herbarium of Chongqing University (voucher number 2010019). Gram-negative (*Escherichia coli *ATCC25922, *Pseudomonas aeruginosa *ATCC27853, *Bacillus proteus *ATCC18663, *Shigella dysenteriae *ATCC18664, *Typhoid bacillus *ATCC18665), Gram-positive (*Staphylococcus aureus *ATCC2925) were offered by Centre for microbial diagnosis of Chongqing Medical University. Penicillin G (sodium salt) was purchased from M&H manufacturing (Samutprakarn, Thailand). Mueller-Hinton Broth (MHB) was purchased from Difco laboratories (Detroit, MI, USA).

(-)-patchouli alcohol was obtained from the National Institute for the Control of Pharmaceutical and Biological Products (Beijing, China). Pogostone was purchased from Sigma Chemicals Co. (St. Louis, MO,USA). All other chemicals used were of analytical grade and purchased from Promega Chemicals Co. (Madison, WI, USA).

*Methods*

*Extract of patchouli oil*

Based on the Chinese Pharmacopoeia 2010 edition, Patchouli oil was extracted as follow: 200g air-dried *Pogostemon cahlin *(Blanco) Benth powder were immersed in water (800 ml) and then heated continuously by use of a fixed power heater with a maximum delivered power of 500W and temperature variation to be ±1 °C. The essential oil which is entrained by azeotropic distillation was freed. The vapour then passed through a condenser outside the power heater where it condensed. The distillate is collected continuously with a Clevenger-type apparatus (14). Condensed water was returned to the flask and heating was continued at 100 °C until no more essential oil was obtained. The essential oil was collected, dried over anhydrous sodium sulphate, and stored at 4 °C until used. 

*Establishment of 3D structure library about compounds in patchouli oil and 5 reference compounds*

**Figure 1:**
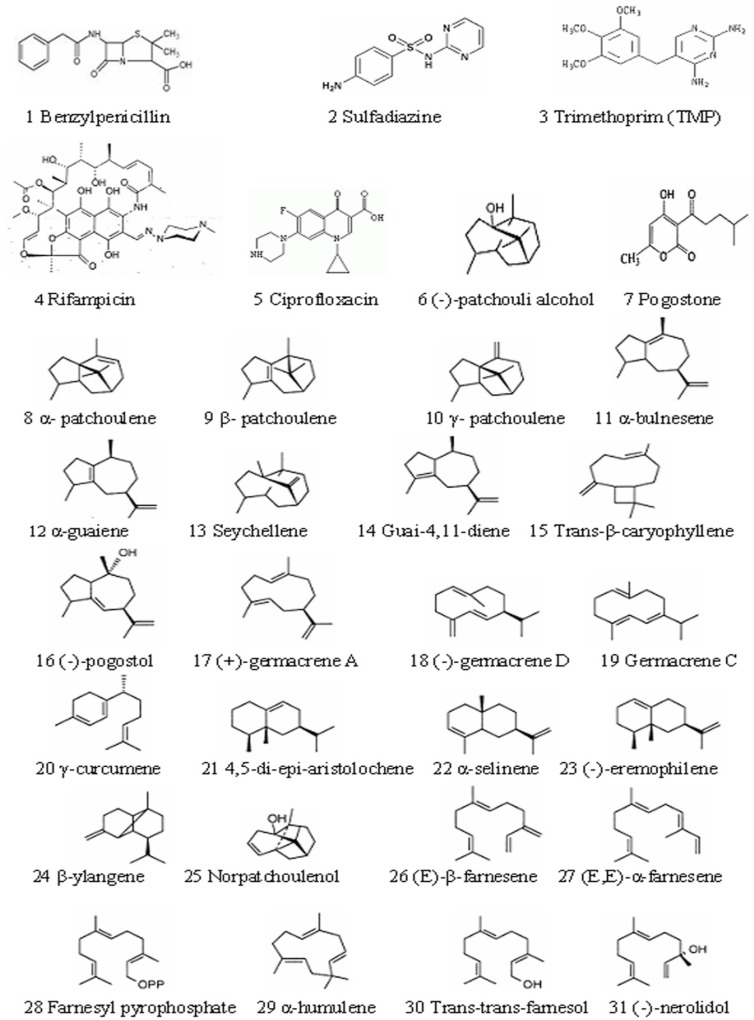
Chemical structures of 5 compared components and 26 chemical compounds in patchouli oil.

The structures of 5 reference compounds and 26 chemical compounds in patchouli oil are shown in Figure 1. (15-17), and their 3D structures were built by using surflex-dock software (8.1).

*Select of five antibacterial targets and their 3D structure*

**Figure 2:**
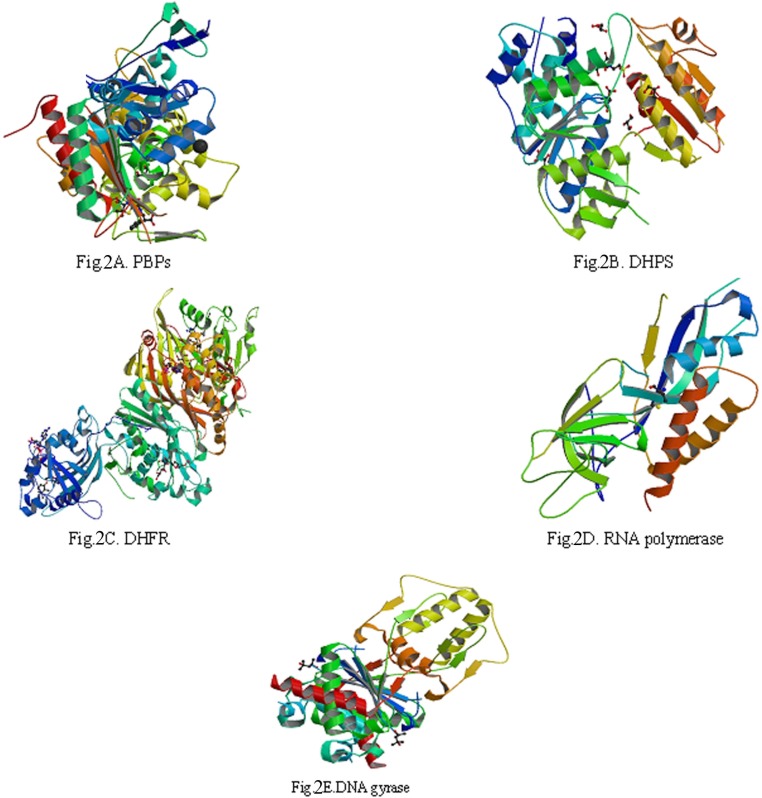
The three-dimensional (3D) structures of 5 enzymes (receptors

5 biological macromolecule enzymes: PBPs (Figure 2A), DHPS (Figure 2B.), DHFR (Figure 2C.), RNA polymerase (Figure 2D.) and DNA gyrase (Figure 2E), which were needed by bacteria in the process of biosynthesis, were selected to serve as target molecules. Their 3D structures were downloaded from PDB which was established in the autumn of 1971 (18-21).

The reason about selection of 5 biological macromolecule enzymes as antibacterial targets were as follows: Cell wall, cell membrane, cell cytoplasm, nucleoplasm (which was aggregated of DNA and RNA, do not have complete nuclear structure, so also called nucleoid) and folic acid were all basic structure of bacterial cell. There were some biosynthetic targets in the structures, and if these targets were combined competitively by drug small molecules, the biosynthesis of a complete bacteria cell structure could not be accomplished, which helped to get antimicrobial effect. In this study, we chose five typical targets such as: 1: Penicillin binding proteins (PBPs, PDB numbered:3OCL) are bacterial enzyme that catalyze the final steps in cell wall biosynthesis and are the lethal targets of *β-*lactam antibiotics such as benzylpenicillin (The 1st chemical composition in Figure 1) , which acted on PBPs and caused bacterial cell walls dissolved (22, 23). 2: DHPS (PDB numberd 3NRS) can promote bacteria folic acid metabolism. Folic acid whose chemical name was “pteroylglutamic acid” was combined with *p*-Aminobenzoic acid (PABA) and glutamic acid *et al*. Under the catalysis of DHPS, Dihydroflic Acid (FAH_2_) are synthetized by PABA and dihydropteridine pyro phosphate. However, FAH_2_ is one of the necessary substances in the process of bacteria combining. Only under exist of folic acid, nucleic acids can be synthesized, and at last the bacteria grow. The drug such as sulfadiazine (the 2^th ^chemical composition in Figure 1) with similar chemical structure of PABA can displace the site of PABA to hinder the folic acid biosynthesis. Finally, bacteria pass away because of lack of folic acid. 3: DHFR (PDB numbered 3INV) is a key enzyme related with folic acid metabolism too. DHFR is a proven target for antibacterial agents, with diaminopyrimidine based inhibitors of DHFR, such as trimethoprim (the 3^th ^chemical composition in Figure 1), used clinically with relative success for decades as a monotherapy and in combination with other agents (24). 4: RNA polymerase (PDB numbered: 2RF4) is essential enzyme involved in protein biosynthesis in bacteria, which have emerged as interesting targets in antibacterial research. RNA polymerase represents a potential drug target. A large number of promising lead compounds have been identified used as the inhibitors of RNA polymerase, Rifampicin (the 4^th^ chemical compound in Figure 1) is known to act upon such a target (an inhibitor of isoleucyl-tRNA synthetase) (25, 26). 5: The bacterial topoisomerases (DNA gyrase and topo IV, with PDB numbered 3M4I and 3LTN, respectively) are multisubunit enzymes that play essential roles in DNA replication and are validated targets for clinically useful antimicrobial drugs. One protein subunit (GyrA or ParC) participates in protein-DNA interactions and other (GyrB or ParE) in ATP hydrolysis. The quinolone antibiotics (*e.g*., ciprofloxacin, the 5th chemical composition in Figure 1) directly affect the gyrase/ topoisomerase- DNA interaction by trapping the proteo-nucleic acid complex at the site of GyrA or ParC (27, 28). 

*Scoring function of surflex-dock*

Molecular docking was carried out between the 31 compounds (ligands) and the 5 enzymes (receptors) by using surflex-dock function. Furthermore, the antibacterial effects of 31 compounds were investigated by scoring function after molecular docking was completed. Surflex- Dock was a software of molecular docking which was used a unique and experiential scoring function and a novel search engine (Based on molecule similarity), which docked ligand molecule to the binding sites of protein (receptor). In other words, the higher experiential scoring function, the better antibacterial effect, so the conformation of complex made from target spot (protein)-chemical components (ligand) was more stable (29). 

*Study on bacteriostatic test in-vitro (Determination of MIC and MBC)*

The minimum inhibitory concentration (MIC) and minimal bactericidal concentration (MBC) was measured by broth dilution method using Mueller-Hinton Broth (MHB) (30, 31).The MIC of patchouli oil, (-)-patchouli alcohol and pogostone were determined turbidimetrically (Genesys 20 spectrophotometer; Thermospectrum), as reported elsewhere (32). In short, patchouli oil, (-)-patchouli alcohol and pogostone were dissolved in MHB. They were adjusted to the desired concentration in a final volume of 20 μL and added into 2 ml of sterile MHB, mixed and serially diluted prior to inoculation with 15 μL of freshly prepared bacteria suspension (107 CFU/mL in MHB). The positive control was performed using 12, 6, 3, 1.5, 0.75, 0.37, 0.18 and 0.09 mM (final concentration) Penicillin G, and blank control tubes contained only MHB and the sample solvent as appropriate. After mixing, the tubes were incubated at 37 °C for 24 h in an incubator (Mermmet model 800). The tubes were then examined after 24 h for visible signs of growth and for turbidity by absorbance at 600 nm. The lowest concentration of each sample that inhibited the bacterial growth was taken as the MIC. The MBC, or the lowest concentration of sample that kills 99.9% of bacteria, was determined by assaying the live organisms of those tubes from the MIC that showed no growth as previously described (30, 32). A loopful of bacterial broth from each of the tubes showing no growth was inoculated onto MHB plates and examined for signs of growth (colonies) after 24 h of incubation at 37°C. All experiments were performed in triplicate.

## Results

*The results of molecular docking*

**Figure 3:**
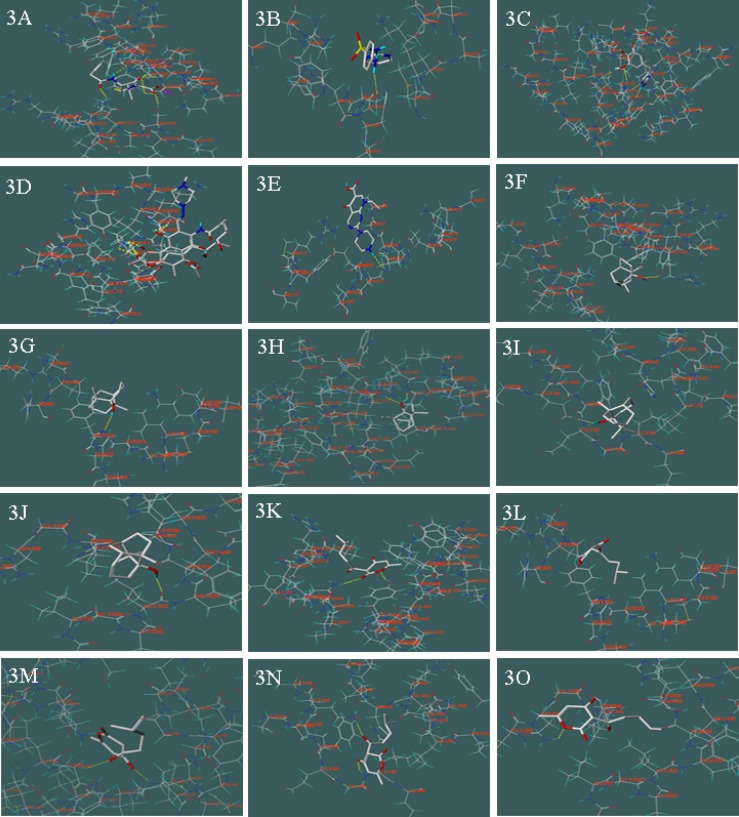
Molecular docking between 31 chemical compounds and 5 enzymes (receptors) respectively; 3A: between benzylpenicillin and PBPs; 3B: between sulfadiazine and DHPS; 3C: between trimethoprim and DHFR; 3D: between rifampicin and RNA polymerase; 3E: between Ciprofloxacin and DNA gyrase; 3F: between (-)-patchouli alcohol and PBPs; 3G: between (-)-patchouli alcohol and DHPS; 3H: between (-)-patchouli alcohol and DHFR; 3I: between (-)-patchouli alcohol and RNA Polymerase; 3J: between (-)-patchouli alcohol and DNA gyrase; 3K: between pogostone and PBPs; 3L: between pogostone and DHPS; 3M: between pogostone and DHFR; 3N: between pogostone and RNA Polymerase; 3O: between pogostone and DNA gyrase

As shown in Table 1. 5 reference compounds, benzylpenicillin, sulfadiazine, trimethoprim, ciprofloxacin and rifampicin, acted on corresponding targets at PBPs (Figure 3A), DHPS (Figure 3B), DHFR (Figure 3C.), RNA Polymerase (Figure 3D), the bacterial topoisomerases (DNA gyrase, Figure 3E), respectively. 

**Table 1:** The scoring function results of 31 chemical constructions

**Number**		**Components (ligands)**	**Target molecules (receptors)**				
			**PBPs**	**DHPS **	**DHFR**	**RNA Polymerase **	**DNA gyrase**
compared components	1	Benzylpenicillin	6.22	-	-	-	-
	2	Sulfadiazine	-	7.33	-	-	-
	3	Trimethoprim	-	-	7.58	-	-
	4	Rifampicin	-	-	-	6.78	-
	5	Ciprofloxacin	-	-	-	-	6.94
The chemical compounds in patchouli oil	6	(-)-patchouli alcohol	6.97	3.97	3.55	4.91	4.22
	7	pogostone	5.02	7.93	5.03	3.77	3.75
	8	*α*- patchoulene	3.56	6.02	4.59	2.92	2.44
	9	*β*- patchoulene	5.45	3.14	6.01	2.03	2.33
	10	*γ*- patchoulene	3.43	6.09	5.07	2.05	2.32
	11	*α*-bulnesene	5.18	5.42	3.45	2.14	3.07
	12	*α*-guaiene	6.23	4.15	5.01	2.96	3.02
	13	Seychellene	3.79	4.54	5.04	2.96	2.59
	14	Guai-4,11-diene	3.41	6.14	3.19	2.98	2.18
	15	Trans-*β*-caryophyllene	3.23	2.58	8.01	3.05	2.37
	16	(-)-pogostol	2.92	7.45	3.12	3.06	2.4
	17	(+)-germacrene A	4.43	5.16	4.33	3.06	4.04
	18	(-)-germacrene D	5.85	5.83	7.73	3.09	2.75
	19	Germacrene C	5.57	5.74	4.25	3.09	2.99
	20	*γ*-curcumene	4.62	2.76	7.05	3.09	3.47
	21	4,5-di-epi-aristolochene	5.47	5.36	4.88	3.13	3.71
	22	*α *–selinene	4.62	4.53	4.21	7.13	2.17
	23	(-)-eremophilene	2.84	2.36	3.27	7.17	2.63
	24	*β*-ylangene	4.25	4.63	8.13	3.18	4.83
	25	Norpatchoulenol	4.68	4.05	5.77	3.18	2.92
	26	(E)-*β*-farnesene	6.28	4.17	3.43	4.81	2.51
	27	(E,E)-*α*-farnesene	4.21	4.61	3.74	3.26	7.04
	28	Farnesyl pyrophosphate	3.22	2.58	3.42	3.28	2.55
	29	*α*-humulene	3.74	3.47	7.79	2.68	4.84
	30	Trans-trans-farnesol	3.98	7.37	3.43	3.26	2.51
	31	(-)-nerolidol	3.98	4.17	4.43	3.52	6.99

Their scorings were 6.22, 7.33, 7.58, 6.78 and 6.94, respectively. In the meantime, 26 compounds in patchouli oil all acted on above 5 targets. To target at PBPs, scoring result of 3 compounds were slightly higher than benzylpenicillin, they were (-)-patchouli alcohol, α-guaiene and (E)-*β*-farnesene, respectively. In these components, (-)-patchouli alcohol scored 6.97. The results showed that they caused bacterial cell walls defects similar to benzylpenicillin. To target DHPS, scoring result of 3 compounds were slightly higher than sulfadiazine, and they were pogostone, (-)-pogostol and Trans-trans- farnesol, respectively. To target at DHFR, scoring result of 4 compounds were slightly higher than trimethoprim, and they were Trans-*β*-caryophyllene, (-)-germacrene D, *β*-ylangene and α-humulene, respectively. The 7 above compounds showed that they can hinder bacteria folic acid metabolism as sulfadiazine or trimethoprim. To target at RNA polymerase, scoring result of 2 compounds were slightly higher than rifampicin, and they were α –selinene and (-)-eremophilene, respectively. The result showed that they can inhibit protein biosynthesis in bacteria. To targets DNA gyrase, scoring result of 2 compounds were slightly higher than ciprofloxacin, and they were (E,E)-*α*-farnesene and (-)-nerolidol, respectively. The result showed that they could inhibit DNA replication in bacteria such as ciprofloxacin.

On the other hand, as shown in Table 1, we found that most of the chemical compounds in patchouli oil were had multi-target effects. We took the (-)-patchouli alcohol (From Figure 3.F to Figure 3.J.) and pogostone (From Figure 3.K. to Figure 3.O.) as examples, that were the most important compounds in patchouli oil. The (-)-patchouli alcohol (with 6.97 scoring on target PBPs, Docking Figure: Figure 3F) mainly caused bacterial cell walls defects as same as benzylpenicillin(with 6.22 scoring on target PBPs). However, we thought that it had other effects on the different targets due to the fact that the scoring result of the (-)-patchouli alcohol with 3.97 (DHPS, Docking Figure Figure 3G), 3.55 (DHFR, Docking Figure: Figure 3H), 4.91(RNA Polymerase, Docking Figure: Figure 3I.) and 4.22 (DNA gyrase, Docking Figure: Figure 3J.), respectively. Similarly, scoring result of the pogostone were 5.02 (PBPs, Docking Figure: Figure 3K.), 7.93 (DHPS, Docking Figure: Figure 3L.), 5.03 (DHFR, Docking Figure: Figure 3M.), 3.77 (RNA Polymerase, Docking Figure: Figure 3N.) and 3.75 (DNA gyrase, Docking Figure: Figure 3O.), respectively. However, on account of 26 compounds in patchouli oil and multi-target effects of each composition, patchouli oil had strong antimicrobial effects, like canister shot. 

*Antimicrobial test in-vitro*

As shown in Table 2. six frequently-used pathogenic bacteria (*Escherichia coli*, *Pseudomonas aeruginosa*, *Bacillus proteus*, *Shigella dysenteriae*, *Typhoid bacillus*, *Staphylococcus aureu*s) were selected for antimicrobial test in vitro, patchouli oil and its two major compounds: (-)-patchouli alcohol and pogostone, whose composition exceeded 60% (g/g) in patchouli oil samples (13), were selected antibacterial agents. The results of MIC and MBC showed that patchouli oil, (-)-patchouli alcohol and pogostone all have good antibacterial activities. These proved that the method and result of molecular docking were feasible and reliable (Table 2). 

**Table 2:** Anti-bacterial activities of patchouli oil and its two chemical compounds

Fungi	Patchouli oil		Patchoulol		Pogostone	
	MIC (mg·mL^-1^)	MBC (mg·mL^-1^)	MIC (mg·mL^-1^)	MBC (mg·mL^-1^)	MIC (mg·mL^-1^)	MBC (mg·mL^-1^)
*Escherichia coli*	4.0	2.0	1.0	2.5	0.45	0.8
*Pseudomonas aeruginosa*	5.5	>10.0	3.5	7.5	3.0	5.5
*Bacillus proteus*	7.5	>10.0	3.5	8.5	4.0	6.5
*Shigella dysenteriae*	6.5	>10.0	3.0	5.0	3.5	6.0
*Typhoid bacillus*	5.5	7.5	6.5	8.0	6.0	>10.0
*Staphylococcus aureus*	4.5	6.5	2.0	7.5	1.0	4.5

## Discussion

The method of molecular docking is primarily used for screening of chemical drugs, is rarely used in applied research on TCM. In this study, 5 biological macromolecule enzymes: PBPs, DHPS, DHFR, RNA polymerase and DNA gyrase, which were necessary for biosynthesis in bacteria, were selected to serve as target molecules. Their 3D structures were downloaded from PDB. Additionally 5 reference antibacterial compounds, benzylpenicillin, sulfadiazine, trimethoprim, rifampicin and ciprofloxacin, which are generally acknowledged as very good antibacterial drugs, were selected for comparison. The 3D structures of the 5 compared components and 26 compounds from patchouli oil were established by using surflex-dock software (8.1). Molecular docking was carried out between the 31 chemical compounds (ligands) and the 5 enzymes (receptors) by using surflex-dock function. Furthermore, the antibacterial effects of 31 chemical compounds were investigated by the scoring function after molecular docking was completed. The results showed that 14 compounds in patchouli oil can well inhibit the bacterial biosynthesis through various means with 5 compared compounds. On the other hand, we found that most compounds in patchouli oil had their multi-target effect. On account of 26 compounds in patchouli oil and multi-target effects of each composition, patchouli oil had strong antimicrobial effects, alike the canister shot which we can call as canister shot-model of multi-target effect. This may be basic principle of TCM therapy. Furthmore, the results of MIC and MBC verified that patchouli oil, (-)-patchouli alcohol and pogostone all have good antibacterial activities. Molecular docking technology and antimicrobial test in vitro all illustrated well the mechanism of action of patchouli oil as antimicrobial drug in TCM therapy. Due to strong antimicrobial effects, particularly potent antimicrobial activity of pogostone and (-)-patchouli alcohol, patchouli oil has broader therapeutic prospects in bacterial infection. 
